# Convalescent Plasma Therapy and Its Effects On COVID-19 Patient Outcomes: A Systematic Review of Current Literature

**DOI:** 10.7759/cureus.9535

**Published:** 2020-08-03

**Authors:** Nabiyah Bakhtawar, Muhammad Usman, Malik Muhammad Uzair Khan

**Affiliations:** 1 Internal Medicine, University Hospital Coventry and Warwickshire, Coventry, GBR; 2 Internal Medicine, Kettering General Hospital, Kettering, GBR; 3 Internal Medicine, Leicester Royal Infirmary, Leicester, GBR; 4 Department of Anatomy, Shalamar Medical and Dental College, Lahore, PAK

**Keywords:** convalescent plasma therapy, convalescent plasma, corona virus, covid 19

## Abstract

Started in late 2019, coronavirus disease 2019 (COVID-19) has rapidly turned into a global pandemic. Considering there is no proven therapy for COVID-19 infection, there is a need to propose potential treatment options. The use of convalescent plasma is one such option as convalescent plasma has previously been used for treating outbreaks of Ebola, influenza, Middle East Respiratory Syndrome Coronavirus (MERS-CoV), and severe acute respiratory (SAR) viruses. Therefore, we carried out an early systematic review to evaluate the efficacy of convalescent plasma (CP) therapy and its effects on COVID-19 patient outcomes. A structured and rigorous systematic review was carried out that included all studies conducted on this topic between December 2019 and June 2020. A total of 10 studies containing a mix of case reports, case series, observational studies, and randomized control trials were identified. Most of the studies lacked randomization and included only small groups of patients. Considering the limitations in the design of current studies, it is difficult to draw a definitive conclusion. However, our results showed that plasma therapy produces notable improvements in patients' clinical symptoms and radiological and biochemical parameters associated with COVID-19 infection. Based on the available information, it is difficult to draw a tangible conclusion about whether plasma therapy improves patient mortality. Until we have concrete evidence to prove otherwise, convalescent plasma therapy may be used as adjuvant therapy for treating COVID-19 infection in critically ill patients.

## Introduction and background

The coronavirus disease 2019 (COVID‐19) has turned into a rapidly evolving pandemic. As of 13th July 2020, the World Health Organization (WHO) has confirmed that the number of COVID-19 cases has reached 12,768,307, and the recorded death toll has crossed 566,654 [1]. WHO estimates that the COVID-19 related mortality curve will level off at 5.7% [2].

Despite the desperate attempts, the treatment for COVID-19 is largely symptomatic. Currently, there are no proven treatments for COVID-19 [3].

Convalescent blood products include whole blood, plasma, serum, and isolates such as immunoglobulins and antibodies. These products are gathered from a patient who has already recovered from an infection and is a possible human source of specific antibodies [4].

Convalescent plasma has previously shown clinical efficacy in other virus-borne infections. WHO recommended the use of convalescent plasma from recovered patients for empirical treatment during the Ebola outbreak [5]. During the 2019 influenza A virus subtype H1N1 pandemic, the use of convalescent plasma therapy by Hung et al. showed a significant reduction in mortality rates in the treatment group compared to control (20.0% vs. 54.8%; p=0.01) [6]. Convalescent plasma therapy has also shown benefit in the treatment of Middle East Respiratory Syndrome Coronavirus (MERS-CoV), and severe acute respiratory infections (SAR) viruses [7, 8]. Several randomized control trials are underway to determine the efficacy of convalescent plasma therapy for COVID-19 infection [9].

There is a lack of structured systematic reviews looking into the efficacy of convalescent plasma therapy for COVID-19 patients. Therefore, we have conducted this early systematic review to provide an insight into the clinical effectiveness of convalescent plasma as a potential therapy for COVID-19 patients.

## Review

Methods

Information Sources

Two independent reviewers (Bakhtawar Nabiyah [BN] and Usman Muhammad [UM]) carried out a literature review using the Preferred Reporting Items for Systematic Reviews and Meta-Analyses (PRISMA) guidelines for a systematic review. This was followed by an independent evaluation of the extracted data by Khan Malik Uzair (KM). We used electronic databases such as PubMed®, Embase®, Google Scholar, Cochrane Library, and MEDLINE® to look for case reports, case series, observational studies, and randomized control trials conducted between December 2019 and June 2020. Two search themes were used for literature review and were joined using the Boolean operator "AND". For the theme "COVID", we used keywords such as "coronavirus", "COVID-19", and "SARS-COV-2". For the theme "convalescent plasma", we used "convalescent plasma" and "plasma therapy" as the main keywords. 

Inclusion Criteria

We included all articles published between December 2019 and June 2020. We included case series, case reports, observational studies, and randomized control trials. We only included full-text manuscripts available in the English language. 

Exclusion Criteria

We excluded review articles, commentaries, notes to editors, and all other articles in which convalescent plasma therapy was not used as a treatment option. We also excluded studies published in languages other than English for which there were no available translated manuscripts. 

Data Extraction and Study Selection

BN and UM carried out a rigorous literature review independently. KM then independently evaluated the results from both the researchers. Once the literature review was complete, the researchers compiled and compared their results for any conflicts that were resolved through mutual consultation. 

A total of 156 studies were identified following the initial literature review. The reviewers used 17 studies after excluding duplicate studies and after reading through the titles, abstracts, and methodologies of the studies. They used 10 studies for their final analysis. 

**Figure 1:**
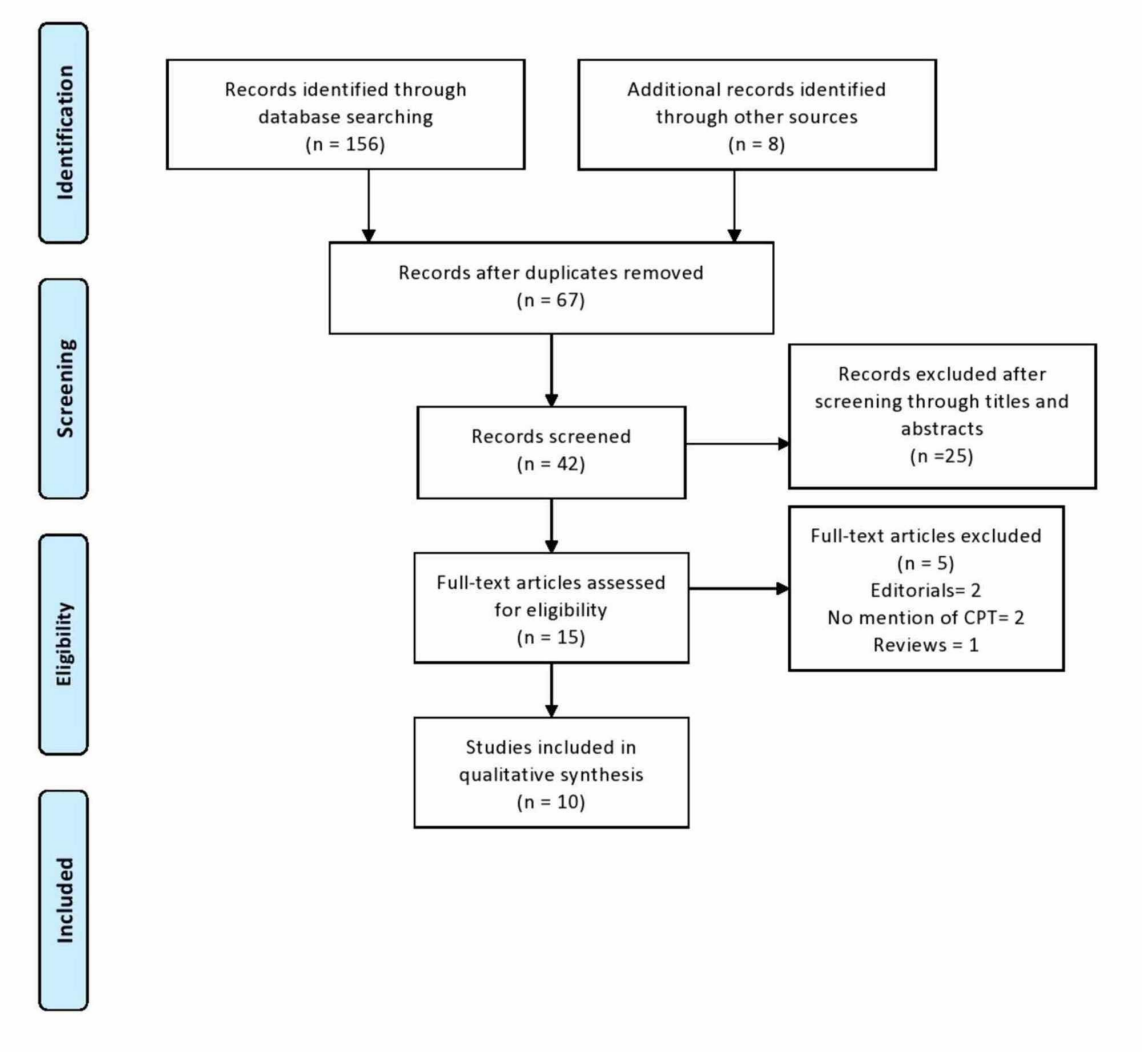
Flowchart describing study identification and selection process CPT - convalescent plasma therapy

Figure 1 describes the literature review process in detail. 

Results

A total of 10 studies were included in this systematic review [10-19]. We were able to identify five case series [10, 12, 13, 15, 16], two case reports [14, 17], one prospective observational study [11], one retrospective observational study [18], and one randomized control trial [19]. These studies included a total of 156 patients with a mean age between 28 and 73 years. Table 1 describes the patient demographics in detail. 

**Table 1:** Summary of study type, patient demographics, plasma therapy intervention, and concurrent treatment modalities PO - by mouth; OD - once daily; BD - twice daily; ECMO - extracorporeal membrane oxygenation

Author	Study type	Number of patients	Median age (years)	Gender	Disease severity	Time of administration of plasma therapy	Intervention used	Concurrent treatment
Ahn et al. 2020 [10]	Case series	2	Male: 71 Female: 67	Male: 1 Female: 1	Severe	Plasma used on day 7 (case 2, female) and day 22 (case 1, male) of presentation	500 ml plasma used in two divided doses	Antiviral therapy: lopinavir/ritonavir 400 mg/100 mg PO BD. Steroids: methylprednisolone 0.5/1 mg/kg/day IV daily. Empirical antibiotics hydroxychloroquine: 400 mg PO OD. Oxygen therapy: intubation and mechanical ventilator care.
Duan et al. 2020 [11]	Prospective observational study	10	52.5	Males: 6 Females: 4	Severe	Between 10 and 20 days of presentation, median administration time 16.7 days	200 ml convalescent plasma with an antibody titer >1:640 given as one dose	Antiviral therapy: ribavirin 0.5 g per day IV, or peramivir 0.3 g per day IV, or oseltamivir 75 mg PO BD, or arbidol 0.2 g PO TDS as a monotherapy or in combination therapy with peramivir 0.3 g per day IV, or remdesivir 0.2 g per day IV, or oseltamivir 75 mg PO BD, or ribavirin 0.5 g per day IV. Steroids: methylprednisolone 20 mg IV daily. Empirical antibiotics and anti-fungals. Oxygen therapy: mechanical ventilation, or oxygenation via high-flow nasal cannula or low-flow nasal cannula.
Pei et al. 2020 [12]	Case series	3 (one patient developed anaphylaxis and dropped out)	Not mentioned	Not mentioned	Moderate to severe	Between 12 and 27 days of hospital admission	200-400 ml antibody titer 1:160 given as one dose	Not mentioned in detail.
Shen et al. 2020 [13]	Case series	5	36-65	Males: 3 Females: 2	Severe	Between 10 and 22 days of admission	200-250 ml plasma with an antibody titer >1:1000 given as two doses	Antiviral therapy: darunavir, ritonavir/lopinavir, arbidol, interferon alfa-1b, or favipiravir. Steroids: methylprednisolone. Empirical antibiotics and anti-fungals. Oxygen therapy: mechanical ventilation.
Tan et al. 2020 [14]	Case report	1	Not mentioned	Male: 1	Moderate	On 48th day of admission	400 ml plasma (doses and antibody titer not mentioned)	Not mentioned in detail.
Ye et al. 2020 [15]	Case series	6	28-75	Males: 3 Females: 3	Severe	One dose given >30 days after admission on average	200 ml plasma given in 1-3 doses (antibody titer not reported)	Antiviral therapy: arbidol. Empirical antibiotics: ofloxacin in one patient. Oxygen therapy.
Zhang et al. 2020 [16]	Case series	4	31-73	Males: 2 Females: 2	Severe	Between 11 and 41 days of admission	Plasma was given in a dose range of 200-2400 mL; given in 1-8 doses (antibody titer not reported)	Antiviral therapy: different anti-virals including lopinavir-ritonavir, interferon-alpha, arbidol, oseltamivir, and ribavirin. Steroids: methylprednisolone. Empirical antibiotics and anti-fungals. Oxygen therapy: mechanical ventilation, high-flow nasal oxygen, ECMO.
Zhang et al. 2020 [17]	Case report	1	64	Female: 1	Severe	On day 17 of hospitalization	200 ml with antibody titer 1:160 (no. of doses not mentioned)	Not mentioned in detail.
Zeng et al. 2020 [18]	Retrospective observational study	21 (treatment group 6, control group 15)	Treatment group: 61.3. Control group: 73	Treatment group: males 5, female 1. control group: males 11, females 4	Severe	Median 21.5 days of hospitalization	300 ml plasma given as two doses to three patients and one dose to three patients	Not mentioned in detail.
Li et al. 2020 [19]	Randomized control trial	103 (treatment group 52, control 51)	Treatment group: 70. Control group: 69	Treatment group: males 27, female 25. Control group: males 33, females 18	Severe or life-threatening COVID-19	Median 27 day of hospitalization	Plasma was given in a dose range of 4 to 13 mL/kg and antibody titer 1:640 (number of doses not clear)	Not mentioned in detail.

All the studies included patients ranging from moderate COVID-19 infection to severe and life-threatening infections. The patients in the studies received plasma therapy between day seven to day 48 of their hospital admission [10-19]. All the studies used varying doses, frequency of administration, and plasma with varying antibody titers. Duan et al. used 200 ml convalescent plasma in one dose [11]. Whereas, Zhang et al. used up to 2,400 ml plasma in up to eight divided doses [16]. Furthermore, most of the studies reported a variety of concurrent treatments such as antivirals, antibiotics, steroids, antimalarial, anti-fungal, and a variety of modalities for oxygen therapy (ranging from the nasal cannula to mechanical ventilation and extracorporeal membrane oxygenation [ECMO]) [10-19] (Table 1). 

Most of the studies reported patient mortalities on follow-up, and almost all patients were alive at the time of follow-up in some studies [10-13, 15-16]. In the study by Zeng at al., five out of six patients died despite receiving plasma therapy [18]. Similarly, Li et al. did not report any difference in mortalities in the treatment vs. control group on the 28th day of follow-up (15.7% vs. 24.0%; p=0.30) [19]. Most of the studies reported a reduction in viral shedding with the viral load turning negative following plasma therapy [10-16, 18-19]. 

The duration of discharge varied from as little as four days following CP therapy to as much as 35 days following CP therapy [13, 15]. However, Li et al. did not report any difference in the time of discharge following CP in treatment vs. control groups (51.0% in treatment vs 36.0% in the control group on day 28 of follow-up; p=0.120) [19].

As for the laboratory parameters, studies showed improvement in C-reactive protein (CRP) [10, 11, 13], interleukin 6 (IL-6) [10, 13], white cell count and/or lymphopaenia [10, 11], procalcitonin [13], and SARS-COV-2 immunoglobulin G (IgG) and immunoglobulin M (IgM) titers [15]. 

Ahn et al. reported a reduction in fever [10], and six studies reported an improvement in the demand for oxygen [10, 11, 13, 15-17]. However, the randomized controlled trials (RCT) by Li et al. did not report any statistically significant difference in clinical improvement in the CP vs. control group on the 28th day of follow-up (51.9% on convalescent plasma group showed clinical improvement vs. 43.1 in the control group; p=0.26) [19]. 

Ahn et al. reported improvement in pulmonary infiltrates as noted on chest X-ray [10]. Three more studies reported improvement in pulmonary infiltrates on repeat CT scans of the chest [11, 15, 16]. 

Table 2 describes the effects of CP therapy on patient outcomes in detail. 

**Table 2:** Table summarizing treatment outcomes following convalescent plasma therapy ICU - intensive care unit, CRP - C-reactive protein; IL-6 - interleukin 6; CP - convalescent plasma; RT-PCR - reverse transcription polymerase chain reaction; PaO2/FiO2 - partial pressure of oxygen in arterial blood/fraction of inspired oxygen.

	All-cause mortality	Duration of discharge from hospital after plasma therapy	Patients discharged from ITU following plasma therapy at the time of follow-up	Improvement in laboratory parameters	Improvement in clinical parameters	Improvement in radiological parameters	Improvement in viral load
Ahn et al. 2020 [10]	Both patients alive at the time of follow-up	18 days	Not reported	Case 1: improvement in CRP and IL-6 to normal. Case 2: improvement in CRP, IL-6, and lymphopenia.	Case 1: fever and oxygen demands subsided. Case 2: significant improvement in oxygen demands.	Case 1: improvement in X-ray pulmonary infiltrates. Case 2: improvement in X-ray pulmonary infiltrates.	Case 1: reduction in SARS-CoV-2 RNA by rRT-PCR. Case 2: complete recovery with no detectable SARS-CoV-2 RNA by rRT-PCR.
Duan et al. 2020 [11]	All patients alive at the time of follow-up	Not reported	Not reported	Reduction in CRP from mean 55.98 before CP therapy to 18.13 after CP therapy; improvement in lymphocytopenia from a mean 0.65 before CP transfusion to 0.76 after therapy.	Improvement in oxygen saturation from mean 93% before CP therapy to 96% after therapy.	CT chest for all patients showed improvement in pulmonary infiltrates following CP therapy.	All patients detected negative for SARS-CoV-2 RNA by rRT-PCR following CP therapy.
Pei et al. 2020 [12]	All patients alive at the time of follow-up	6, 14, 23 days for three patients	All discharged	Not reported	Not clearly mentioned	Not reported	Two patients had negative viral load as detected via SARS-CoV-2 nucleic acid test after CP therapy, third patient developed anaphylaxis and dropped out.
Shen et al. 2020 [13]	All patients alive at the time of follow-up	32, 33, 35 days for three patients (only three patients followed)	Not clear, probably three discharged	CRP, Il-6, and procalcitonin levels dropped significantly on day 12 post-transfusion.	PAO2/FIO2 ranged from 172-276 pre-transfusion and improved to 284-366 on the day 12 post-transfusion Body temperature ranged from 37.6-39.0°C pre-transfusion and became normal on the third day post-transfusion.	Not reported	CT value became negative for all patients on day 12 post-transfusion,
Tan et al. 2020 [14]	Not reported	Not reported	Probably all discharged	Not reported	Not reported	Not reported	Oropharyngeal swab became negative on the fourth day of transfusion.
Ye et al.2020 [15]	All patients alive at the time of follow-up	4, 6, 6, 10 for four patients (unclear for one patient)	Five discharged	Improvement in SARS-COV-2 IgM and IgG titer following CP therapy.	5/6 patients reported improvement in shortness of breath and oxygen requirements.	Resolution of ground glass opacifications for 5/6 patients on repeat CT scans following CP therapy.	Throat COVID swabs negative for 5/6 patients following CP therapy
Zhang et al. 2020 [16]	All patient alive at the time of follow-up, one patient in ICU	7, 25, 27 (three patients followed)	Three discharged	Not reported	Improvement in oxygen saturation.	Significant improvement in pulmonary infiltrates noted on repeat imaging (chest radiographs and CT scans).	RT-PCR and oropharyngeal swabs noted to be negative.
Zhang et al. 2020 [17]	Not reported	Not reported	Probably all discharged	Not reported	Improvement in ventilation status with patient not requiring mechanical ventilation on day 11 of CP therapy.	Not reported	Not reported
Zeng et al. 2020 [18]	Five patients died. No changes in mortality noted with the use of CP.	Not reported	One discharged	Not reported	Not reported	Not reported	RT-PCR and oropharyngeal swabs noted to be negative for all patients.
Li et al. 2020 [19]	No statistically significant difference in 28-day mortality in treatment vs. control group (15.7% vs 24.0%; p=0.30),	No statistically significant difference time to discharge on day 28 of follow up (51.0% in treatment vs 36.0% in the control group p=0.12)	21/23 (91.3) and 15/22 (68.2) patients discharged in the treatment and control group respectively on day 28 of follow up.	Not reported	No statistically significant clinical improvement achieved on day 28 of follow-up (51.9% [27/52] patients improved the convalescent plasma group vs 43.1% (22/51) in the control group; p=0.26).	Not reported	SARS-CoV-2 viral PCR reported negative earlier compared to the control group (87.2% treatment group vs 37.5% control group; p<0.001 .

Discussion

The randomized evaluation of COVID-19 therapy (RECOVERY) trial is the only large scale trial suggesting dexamethasone as an effective treatment for reducing COVID-19 mortality in critically ill patients [20]. Despite the acceleration of the COVID-19 spread, we are still struggling to find a concrete treatment. Therefore, our systematic review is valuable as it explores the current literature and aims at assessing the efficacy of convalescent plasma therapy for treating COVID-19. 

Plasma therapy has long been used for the treatment of infectious diseases such as Ebola, MERS, and SARS [5-8]. Schoofs et al. suggested that antibodies in convalescent plasma suppresses viremia and tested 3BNC117 antibody for its ability to suppress HIV-1 viremia. 3BNC117 is a potent antibody that binds to the CD4 binding sites on the viral envelope. Even after a single passive administration in animal models, Schoof et al. noted the antibody to suppress HIV-1 viremia [20]. In-vivo studies also suggest that antibodies not only reduce the viral load and reduce the rate of infection of new cells but increase the clearance rate of existing infected cells as well [21]. 

Our systematic review noted that there was no standardization in terms of the time of administration of plasma therapy. Existing research suggests that SARS viral viremia peaks during the first week of infection and patients usually start to develop primary immune response by the end of the second week of their infection. Therefore, the administration of plasma early during the early stage of the disease might lead to more favorable clinical outcomes [22]. 

Most of the studies included in our systematic review showed that convalescent plasma therapy leads to an improvement in clinical outcomes. However, the only RCT by Li et al. showed that the patients receiving CP did not differ from control groups on the six-point clinical severity scale on the 28th day of follow-up [19]. Furthermore, almost all patients were discharged in the rest of the studies by the only RCT by Li et al. noted that the mortality did not change significantly between CP and control groups [19]. 

Limitations 

The results of the available research should be interpreted with great caution. The available data suggesting positive effects of CP on patients’ clinical symptoms and mortality mainly come from case reports and case series that lack randomization, have a limited data set, and have a high risk of bias. The only available RCT suggests otherwise and does not report any changes in mortality and improvement in clinical symptoms with the use of CP. Furthermore, it must also be noted that the use of convalescent plasma for COVID-19 has significant clinical and practical limitations. As noted in previous studies, patients recovering from SARS infection require at least 12 weeks for their IgG neutralizing antibody titer (NAT) to reach ≥1:160 and only the CP that had a NAT of ≥1:160 reduced mortality in SARS cases [23]. Moreover, limitations such as getting informed consent from the donors and recipients, state of health of donor and recipient, the amount of plasma acquired from one donor, and the mismatch of the number of donors versus the patients who need this therapy may significantly limit the clinical utility of CP for treating COVID-19 cases [24]. Also, adverse reactions such as transfusion-related anaphylactic reactions, the transmission of infections, and other adverse events such as fever, chills, and lung injury are valid clinical concerns that should not be overlooked [25]. 

## Conclusions

COVID-19 is a global pandemic with no proven treatment. The changing situation is posing a serious therapeutic dilemma for the clinicians and there is an urgent need for therapies that could help reduce patient mortality. Amidst the therapeutic uncertainties, convalescent plasma therapy might have some therapeutic potential. Our systematic review shows that plasma therapy might produce a notable improvement in patient symptoms and clinical and biochemical parameters associated with COVID-19 infection. Although there is some preliminary evidence that plasma therapy might improve patient mortality but this fact needs to be validated through organized RCTs. Despite the potential benefits, plasma therapy has significant limitations such as lack of availability, a dearth of standardization of this treatment method, and paucity of compelling clinical evidence advocating its use. Despite these limitations, the early use of convalescent plasma therapy may be considered as an adjuvant for critically-ill COVID-19 patients. 
